# Severe Hypercalcemia During Lactation in a Patient With Pre-existing Hypoparathyroidism

**DOI:** 10.7759/cureus.112172

**Published:** 2026-07-06

**Authors:** Amit K Sumal, Shalini Bhat

**Affiliations:** 1 Endocrinology, Diabetes and Metabolism, University of California Los Angeles, Los Angeles, USA; 2 Endocrinology, Diabetes and Metabolism, Veterans Affairs (VA) Greater Los Angeles Healthcare System, Los Angeles, USA

**Keywords:** calcium, high-risk pregnancy, hypercalcemia, lactation, parathyroid hormone-related peptide (pthrp), post-surgical hypoparathyroidism

## Abstract

Calcium homeostasis undergoes significant physiologic changes during pregnancy and lactation to support fetal skeletal development and neonatal calcium needs. During pregnancy, calcium requirements are met by increased activation of 1,25-dihydroxyvitamin D (1,25-(OH)_2_D) by placental 1a-hydroxylase and by increased maternal and placental secretion of parathyroid hormone-related protein (PTHrP). Conversely, during lactation, calcium demand is met by maternal bone resorption driven by postpartum decline in estrogen levels and by PTHrP produced by maternal breast tissue. These physiologic adaptations complicate the management of patients with primary hypoparathyroidism, who require calcium and calcitriol supplementation to maintain eucalcemia. Herein, we describe a case of a 35-year-old woman with post-operative hypoparathyroidism following total thyroidectomy for metastatic papillary thyroid carcinoma who developed hypercalcemia during lactation after each of her two pregnancies. Reducing her calcium and calcitriol supplementation rapidly normalized her calcium levels. This case highlights how physiologic changes in calcium metabolism during pregnancy and lactation can significantly alter calcium and calcitriol requirements in patients with hypoparathyroidism. Continuation of prepartum calcium and calcitriol regimens during pregnancy and lactation can cause maternal hypercalcemia. Thus, close surveillance of calcium levels in women with hypoparathyroidism is essential to adjust calcium and calcitriol supplementation and avoid hypercalcemia.

## Introduction

Calcium homeostasis undergoes significant changes during pregnancy and lactation. During pregnancy, 30 g of calcium is required to build the fetal skeleton, with the greatest demand in the third trimester [1]. These needs are met by two mechanisms. First, placental 1a-hydroxylase increases 1,25-dihydroxyvitamin D (1,25-(OH)_2_D) production fivefold, doubling maternal intestinal calcium absorption to 400 mg per day. Secondly, increased secretion of placental and fetal parathyroid hormone-related protein (PTHrP) along with increased 1,25 (OH)_2_D levels increases both bone resorption and renal calcium conservation and promotes calcium transfer from mother to fetus [1,2].

In contrast, during lactation, the neonate’s calcium requirements are met through calcium in breastmilk. Sufficient calcium levels in breastmilk are achieved by maternal bone resorption from the decline in estrogen levels postpartum and PTHrP production from maternal breast tissue [1,2]. This increase in bone turnover releases 300-1,000 mg of calcium daily, and maternal spine and hip density can decrease by 10% during the first six months of lactation [1-3]. Furthermore, 1,25-(OH)_2_D levels slowly normalize from their increased levels during pregnancy [1,2].

Primary hypoparathyroidism is characterized by insufficient parathyroid hormone (PTH) production and results in hypocalcemia that often requires calcium and calcitriol supplementation. However, peripartum changes in calcium metabolism complicate the treatment of primary hypoparathyroidism and lead to markedly variable calcium and calcitriol requirements to maintain eucalcemia [2,4-6]. In this case, we describe a patient with hypoparathyroidism who subsequently developed hypercalcemia during lactation after each of her two pregnancies. This case highlights the physiologic adaptations in calcium homeostasis during pregnancy and lactation and underscores the importance of monitoring calcium levels to adjust calcium and calcitriol supplementation and prevent hypercalcemia. This article was previously presented as a meeting abstract at the 2022 ENDO Endocrine Society Meeting on June 11-14, 2022. The abstract was published in the *Journal of the Endocrine Society* on November 1, 2022.

## Case presentation

A 35-year-old woman with a history of post-operative hypoparathyroidism after total thyroidectomy for metastatic papillary thyroid carcinoma was found to have hypercalcemia during lactation, two months after delivery of her first pregnancy. She was taking calcitriol 0.5 mcg two times daily prepartum, which she continued throughout pregnancy and postpartum. Two weeks before delivery, her serum calcium was 9.7 mg/dL (n: 8.6-10.3 mg/dL). However, testing two months postpartum revealed markedly elevated serum calcium 13.7 mg/dL and ionized calcium 1.70 mmol/L (n: 1.09-1.29 mmol/L). Subsequent workup was notable for low parathyroid hormone (PTH) 4 pg/mL (n: 11-51 pg/mL), and normal PTHrP 2.8 pg/mL (n: <14-27 pg/mL), 25-hydroxyvitamin D (25-(OH)D) 60 ng/mL (n: 30-80 ng/mL), and 1,25-(OH)_2_D 38.0 pg/mL (n: 19.9-79.3 pg/mL) (Table 1). Her calcitriol was reduced to 0.25 mcg once daily, and her serum and ionized calcium improved to 10.2 mg/dL and 1.18 mmol/L, respectively, within 10 days.

**Table 1:** Trend of Laboratory Markers Before, During, and After Both Pregnancies 1,25-(OH)_2_D = 1,25-Dihydroxyvitamin D, 25-(OH)D = 25-Hydroxyvitamin D, PTH = Parathyroid Hormone, PTHrP = Parathyroid Hormone-Related Protein. Notable laboratory values are in bold.

Lab Test	Pregnancy #1			Pregnancy #2			Reference Range
	Prepartum	Two Weeks Prior to Delivery	Two Months Postpartum During Lactation	Prepartum	One Month Prior to Delivery	Two Months Postpartum During Lactation	
Serum Calcium (Serum Calcium Corrected for Albumin)	10.2 (9.7) mg/dL	9.7 (9.9) mg/dL	13.7 (13.3) mg/dL	10.1 (9.4) mg/dL	8.0 (8.5) mg/dL	10.4 (10.2) mg/dL	8.6-10.4 mg/dL
Ionized Calcium	1.26 mmol/L		1.70 mmol/L	1.24 mmol/L	1.04 mmol/L	1.31 mmol/L	1.09-1.29 mmol/L
PTH	5 pg/mL		4 pg/mL	4 pg/mL	5 pg/mL	5 pg/mL	11-51 pg/mL
PTHrP			2.8 pg/mL	13 pg/mL	22 pg/mL	17 pg/mL	14-27 pg/mL
25-(OH)D	69 ng/mL		60 ng/mL	47 ng/mL	49 ng/mL	47 ng/mL	20-50 ng/mL
1,25-(OH)_2_D	61.0 pg/mL		38.0 pg/mL	60.2 pg/mL	108.0 pg/mL	70.2 pg/mL	15-75 pg/mL

Two years later, after her second pregnancy, she again developed hypercalcemia during lactation. One month before delivery, her serum calcium was 8.0 mg/dL and her ionized calcium was 1.04 mmol/L on a regimen of calcium carbonate 1,000 mg three times daily and calcitriol 0.5 mcg twice daily. However, two months after delivery and during lactation, her serum and ionized calcium increased to 10.4 mg/dL and 1.31 mmol/L, respectively. Evaluation of her PTHrP levels revealed an increase from 13 pg/mL one year prepartum to 22 pg/mL during her third trimester, and then to 17 pg/mL two months postpartum. In addition, her 1,25-(OH)_2_D increased from 60.2 pg/mL one year prepartum to 108.0 pg/mL during her third trimester and decreased to 70.2 pg/mL two months postpartum (Table 1). Reducing her calcium and calcitriol supplementation again improved her serum and ionized calcium levels.

## Discussion

This case illustrates the differences in calcium metabolism during pregnancy and lactation and how these shifts can significantly change calcium and calcitriol doses in patients with primary hypoparathyroidism. During pregnancy, both PTHrP and 1,25-(OH)_2_ increase to satisfy fetal calcium requirements (Table 1). PTHrP and 1,25-(OH)_2_ increase bone resorption and renal calcium conservation, and 1,25-(OH)_2_ additionally increases intestinal calcium absorption. However, during lactation, 1,25-(OH)_2_ levels decrease, and calcium requirements in breastmilk are instead achieved by maternal breast production of PTHrP and maternal bone resorption from postpartum decline in estrogen (Table 1). In this case, these physiologic changes resulted in severe hypercalcemia during lactation after both of her pregnancies.

Close monitoring of calcium levels to guide calcium and calcitriol replacement during pregnancy and lactation is essential to avoid hypercalcemia in patients with hypoparathyroidism. However, clear consensus guidelines for the management of hypoparathyroidism during pregnancy remain elusive. Case series and reports provide much of the data on peripartum hypoparathyroidism management [2,4,5]. Although one paper offers evidence-based guidance, the overall quality of evidence remains low to very low due to limited data [6].

While many case reports indicate decreased calcium and calcitriol needs during pregnancy, some have instead reported increased requirements [4,5]. This variability may be linked to differences in patients' dietary calcium intake [6]. Therefore, it is important to carefully monitor calcium levels in pregnant patients with hypoparathyroidism to adjust calcium and calcitriol supplementation appropriately. Additionally, serum calcium can appear artificially low during pregnancy due to intravascular volume expansion, which causes hemodilution and reduces serum albumin levels. As a result, albumin-corrected calcium and ionized calcium serve as more reliable indicators of calcium status and should be measured every three to four weeks during pregnancy [2,6].

Although calcium and calcitriol requirements can vary during pregnancy, lower doses are typically warranted postpartum to prevent hypercalcemia during lactation, as shown in this case. One case report even describes discontinuing calcium and calcitriol supplementation entirely during lactation [7]. However, there are no specific recommendations on reducing calcium and calcitriol doses during lactation. Current recommendations are to monitor serum albumin-corrected or ionized calcium measurements every four to six weeks during lactation [2,6].

In patients with hypoparathyroidism, albumin-corrected or ionized calcium levels should be maintained within the low-normal range during pregnancy and lactation [6]. It is essential to avoid hypocalcemia to prevent compensatory fetal hyperparathyroidism, which can lead to demineralization of the fetal skeleton [8]. Maternal hypocalcemia has also been associated with miscarriage, preterm labor, and stillbirth [9]. Conversely, hypercalcemia should be avoided to prevent intrauterine growth restriction, premature birth, and low birth weight [10]. Maternal hypercalcemia can also suppress the fetal parathyroid glands, potentially causing fetal hypocalcemia, tetany, and rarely, parathyroid aplasia [11]. As demonstrated in this case, because calcitriol has a half-life of four to six hours, hypercalcemia can be corrected quickly in cases of overtreatment.

**Figure 1:**
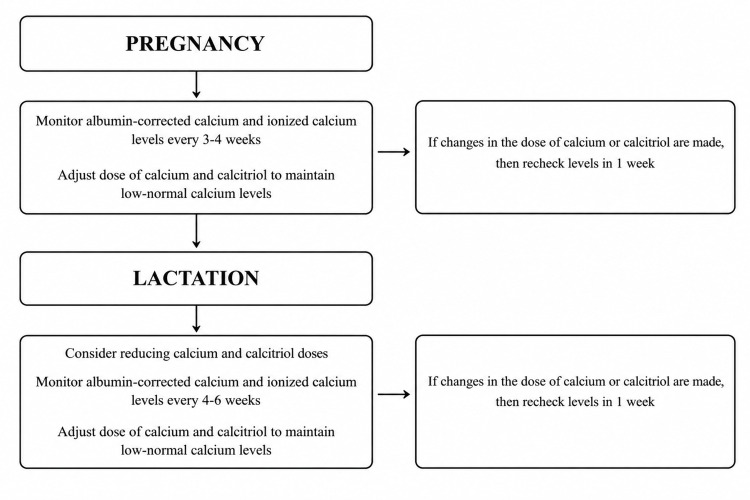
Proposed Management Algorithm for Patients With Hypoparathyroidism During Pregnancy and Lactation

We have synthesized both our experience from this case and current recommendations to propose a management algorithm for patients with hypoparathyroidism during pregnancy and lactation (Figure 1). In alignment with current guidelines, we recommend monitoring albumin-corrected calcium and ionized calcium every three to four weeks during pregnancy and every four to six weeks during lactation [2,6]. If changes in the dose of calcium or calcitriol are made, then we recommend rechecking levels in one week. During lactation, an empiric reduction in the doses of calcium and calcitriol should be considered to avoid hypercalcemia. Calcium and calcitriol doses should be adjusted to maintain albumin-corrected and ionized calcium levels within the low-normal range.

## Conclusions

We describe a case of hypercalcemia in a woman with pre-existing hypoparathyroidism following her two pregnancies during lactation. This case highlights peripartum changes in calcium homeostasis, which can significantly alter calcium and calcitriol requirements in women with primary hypoparathyroidism. As illustrated in this case, continuation of prepartum calcium and calcitriol regimens during pregnancy and lactation can cause maternal hypercalcemia. Thus, close surveillance of calcium levels peripartum in women with hypoparathyroidism is crucial to adjust calcium and calcitriol supplementation and maintain adequate calcium levels in both mother and child.

## References

[1] Kovacs CS (2016). Maternal mineral and bone metabolism during pregnancy, lactation, and post-weaning recovery. Physiol Rev.

[2] Tsourdi E, Anastasilakis AD (2021). Parathyroid disease in pregnancy and lactation: a narrative review of the literature. Biomedicines.

[3] Kyvernitakis I, Reuter TC, Hellmeyer L, Hars O, Hadji P (2018). Subsequent fracture risk of women with pregnancy and lactation-associated osteoporosis after a median of 6 years of follow-up. Osteoporos Int.

[4] Hatswell BL, Allan CA, Teng J (2015). Management of hypoparathyroidism in pregnancy and lactation: a report of 10 cases. Bone Rep.

[5] Callies F, Arlt W, Scholz HJ, Reincke M, Allolio B (1998). Management of hypoparathyroidism during pregnancy: report of twelve cases. Eur J Endocrinol.

[6] Khan AA, Clarke B, Rejnmark L, Brandi ML (2019). Management of endocrine disease: hypoparathyroidism in pregnancy: review and evidence-based recommendations for management. Eur J Endocrinol.

[7] Mather KJ, Chik CL, Corenblum B (1999). Maintenance of serum calcium by parathyroid hormone-related peptide during lactation in a hypoparathyroid patient. J Clin Endocrinol Metab.

[8] Landing BH, Kamoshita S (1970). Congenital hyperparathyroidism secondary to maternal hypoparathyroidism. J Pediatr.

[9] Eastell R, Edmonds CJ, de Chayal RC, McFadyen IR (1985). Prolonged hypoparathyroidism presenting eventually as second trimester abortion. Br Med J (Clin Res Ed).

[10] Scholl TO, Chen X, Stein TP (2014). Maternal calcium metabolic stress and fetal growth. Am J Clin Nutr.

[11] Cemeroglu AP, Böber E, Büyükgebiz A (2001). Prolonged hypocalcemia in a 2 month-old boy unmasking maternal diagnosis of primary hyperparathyroidism. J Pediatr Endocrinol Metab.

